# LIPG endothelial lipase and breast cancer risk by subtypes

**DOI:** 10.1038/s41598-021-89669-4

**Published:** 2021-05-17

**Authors:** Manuela Gago-Dominguez, Carmen M. Redondo, Manuel Calaza, Marcos Matabuena, Maria A. Bermudez, Roman Perez-Fernandez, María Torres-Español, Ángel Carracedo, J. Esteban Castelao

**Affiliations:** 1 Galician Public Foundation of Genomic Medicine (FPGMX), Servicio Galego de Saúde (SERGAS), Santiago de Compostela, Spain; 2 Genomic Medicine Group, Center for Research in Molecular Medicine and Chronic Diseases (CIMUS), Centro en Red de Enfermedades Raras (CIBERER), University of Santiago de Compostela, Santiago de Compostela, Spain; 3 Galician Public Foundation of Genomic Medicine (FPGMX), Genomic Medicine Group, International Cancer Genetics and Epidemiology Group, Health Research Institute of Santiago (IDIS), Santiago de Compostela, Spain; 4 Oncology and Genetics Unit, Instituto de Investigación Sanitaria Galicia Sur, Vigo, Spain; 5 Conselleria de Educación, Xunta de Galicia, Santiago de Compostela, Spain; 6 Centro de Investigación en Tecnoloxías da Información (CiTIUS), University of Santiago de Compostela, Santiago de Compostela, Spain; 7 Department of Biology, Faculty of Science, University of A Coruña, A Coruña, Spain; 8 Department of Physiology and Center for Research in Molecular Medicine and Chronic Diseases (CIMUS), University of Santiago de Compostela, Santiago de Compostela, Spain

**Keywords:** Breast cancer, Predictive markers

## Abstract

Experimental data showed that endothelial lipase (LIPG) is a crucial player in breast cancer. However, very limited data exists on the role of LIPG on the risk of breast cancer in humans. We examined the LIPG-breast cancer association within our population-based case–control study from Galicia, Spain, BREOGAN (BREast Oncology GAlicia Network). Plasma LIPG and/or OxLDL were measured on 114 breast cancer cases and 82 controls from our case–control study, and were included in the present study. The risk of breast cancer increased with increasing levels of LIPG (multivariable OR for the highest category (95% CI) 2.52 (1.11–5.81), P-trend = 0.037). The LIPG-breast cancer association was restricted to Pre-menopausal breast cancer (Multivariable OR for the highest LIPG category (95% CI) 4.76 (0.94–28.77), P-trend = 0.06, and 1.79 (0.61–5.29), P-trend = 0.372, for Pre-menopausal and Post-menopausal breast cancer, respectively). The LIPG-breast cancer association was restricted to Luminal A breast cancers (Multivariable OR for the highest LIPG category (95% CI) 3.70 (1.42–10.16), P-trend = 0.015, and 2.05 (0.63–7.22), P-trend = 0.311, for Luminal A and non-Luminal A breast cancers, respectively). Subset analysis only based on HER2 receptor indicated that the LIPG-breast cancer relationship was restricted to HER2-negative breast cancers (Multivariable OR for the highest LIPG category (95% CI) 4.39 (1.70–12.03), P-trend = 0.012, and 1.10 (0.28–4.32), P-trend = 0.745, for HER2-negative and HER2-positive tumors, respectively). The LIPG-breast cancer association was restricted to women with high total cholesterol levels (Multivariable OR for the highest LIPG category (95% CI) 6.30 (2.13–20.05), P-trend = 0.018, and 0.65 (0.11–3.28), P-trend = 0.786, among women with high and low cholesterol levels, respectively). The LIPG-breast cancer association was also restricted to non-postpartum breast cancer (Multivariable OR for the highest LIPG category (95% CI) 3.83 (1.37–11.39), P-trend = 0.003, and 2.35 (0.16–63.65), P-trend = 0.396, for non-postpartum and postpartum breast cancer, respectively), although we lacked precision. The LIPG-breast cancer association was more pronounced among grades II and III than grade I breast cancers (Multivariable ORs for the highest category of LIPG (95% CI) 2.73 (1.02–7.69), P-trend = 0.057, and 1.90 (0.61–6.21), P-trend = 0.170, for grades II and III, and grade I breast cancers, respectively). No association was detected for OxLDL levels and breast cancer (Multivariable OR for the highest versus the lowest category (95% CI) 1.56 (0.56–4.32), P-trend = 0.457).

## Introduction

The triglyceride lipase gene (TLG) family includes secreted lipases that hydrolyze triglycerides and phospholipids. The resulting fatty acids are taken up by the surrounding tissue in which they contribute to the intracellular fatty acid pool after incorporation into cellular lipids. The most well known members of the TLG family are endothelial lipase (LIPG or EL)^1,2^, lipoprotein lipase (LPL)^3^, hepatic lipase (HL)^4^, and pancreatic lipase (PL)^5^.

LIPG has primarily a phospholipase activity, also a low triglyceride lipase activity, and an important role in plasma high density lipoproteins (HDL) metabolism^6–10^. The main substrate of LIPG are phospholipids from HDL^11^. Several studies have shown that LPL plays an important role in carcinogenesis, including colorectal, pancreatic, and lung cancers^11,12^. However, LIPG has not been reported to be associated with any cancer except testicular germ cell tumors^13^ and gastric cancer^14^.

Experimental data have shown LIPG to be a crucial player in breast cancer since it provides the indispensable extracellular lipids needed for breast cancer cells to grow^15^. However, no data exist on the role of LIPG on the risk of breast cancer in humans. Oxidized Low Density Lipoprotein (OxLDL) is a marker of oxidative stress. A previous study shows inverse association with breast cancer risk^16^.

Using data from the Breast Oncology Galician Network Study (BREOGAN), we will examine the effect of plasma endothelial lipase (LIPG) and OxLDL on the risk of breast cancer, overall and by major subtypes, as well as menopausal status, time of diagnosis, grade and morphology, in Spanish women.

## Results

Characteristics of study cases and controls have been previously described^17–21^. Table 1 present the characteristics of patients and controls of the present study. Briefly, 25% of cases reported having a family history of breast or ovarian cancer, and 31% of cases reported having used oral contraceptives. The corresponding figures among controls were 13% and 56%. Eleven percent of the cases were nulliparous and 70% of cases had menarche after age 12. The corresponding figures among controls were 27% and 78%, respectively. Cases were similar to non-cases on all other characteristics such as hormonal factors, BMI, and HRT use. Consistent with previous studies, invasive ductal carcinoma was the most frequent histological type (74%), and the distribution of the Luminal A, Luminal B, HER2 overexpressed, and TNBC subtypes were 65%, 10%, 3% and 7%, respectively. Mean tumor size in cm was 2.0.

**Table 1:** Associations between risk/protective factors for breast cancer and breast cancer risk.

	Cases N (%)	Controls N (%)	OR^a^	95% CI	OR^b^	95% CI
N	114	82				
Mean age (years)	57.0 ± 13.4	52.2 ± 15.4				
**Age at menarche (years)**						
≤ 12	32	18	1		1	
> 12	76	64	0.66	0.33–1.27	0.68	0.33–1.37
**Age at menopause (years)**						
> 50	22	13	1		1	
≤ 50	33	18	1.07	0.43–2.63	0.98	0.38–2.50
**Family history**^**c**^						
No	85	71	1		1	
Yes	29	11	2.12	1.01–4.75	2.70	1.19–6.53
**Number of pregnancies**						
0	13	22	1		1	
1–2	74	41	2.58	1.16–5.91	2.27	0.97–5.44
≥ 3	26	19	1.51	0.54–4.25	1.46	0.49–4.42
P-trend				0.456		0.747
**Body mass index (kg/m**^**2**^**)**						
< 25	48	44	1		1	
25–29	34	19	1.47	0.73–3.02	2.27	1.04–5.12
≥ 30	32	19	1.17	0.55–2.50	1.48	0.64–3.50
P-trend				0.792		0.499
**Oral contraceptive use**						
Never	35	18	1		1	
Ever	16	23	0.37	0.15–0.89	0.41	0.16–1.04
**Hormone replacement therapy**						
Never	49	37	1		1	
Ever	3	4	0.52	0.10–2.54	0.70	0.11–3.94
**Histology type**						
Ductal	84 (73.7)					
Lobular	6 (5.3)					
Mucinous	3 (2.6)					
Mixed	3 (2.6)					
Other	2 (1.7)					
**Tumor size (cm)**	2.0 (1.3)					
**Breast cancer subtypes**						
Luminal A	74 (64.9)					
Luminal B	12 (10.5)					
TNBC	8 (7.0)					
HER2 overexpressed	4 (3.5)					

^a^Adjusted for age at diagnosis (cases) and age at interview (controls).

^b^Further adjusted for age at menarche, parity, menopausal status, BMI, and family history.

^c^Defined as one or more first and/or second-degree relatives with breast and/or ovarian cancer.

Table 2 shows breast cancer risk in relation to levels of LIPG and OxLDL. Breast cancer risk increased with increasing levels of LIPG (Multivariable OR for the highest category (95% CI) 2.52 (1.11–5.81), P-trend = 0.037). No association was detected for OxLDL levels and breast cancer (Multivariable OR for the highest versus the lowest category (95% CI) 1.56 (0.56–4.32), P-trend = 0.457).

**Table 2:** LIPG and Oxidized LDL and breast cancer risk.

	Number cases/controls	OR^a^	95% CI	OR^b^	95% CI
LIPG < 1.18 ng/ml	23/23	1.00		1.00	
LIPG 1.18–2.78 ng/ml	21/23	0.89	0.38–2.05	0.88	0.35–2.24
LIPG > 2.78 ng/ml	63/23	2.68	1.27–5.76	2.52	1.11–5.81
P-trend			0.009		0.037
OxLDL < 68.34 UL	25/16	1.00		1.00	
OxLDL 68.34–94.14 UL	35/15	1.30	0.52–3.23	1.70	0.61–4.88
OxLDL > 94.14 UL	40/16	1.16	0.46–2.88	1.56	0.56–4.32
P-trend			0.656		0.457

^a^Adjusted for age at diagnosis (cases) and age at interview (controls).

^b^Further adjusted for age at menarche, parity, menopausal status, BMI, and family history of breast/ovarian cancer.

Table 3 presents the association between LIPG and breast cancer risk according to menopausal status. The LIPG-breast cancer association was restricted to Pre-menopausal breast cancer (Multivariable OR for the highest category (95% CI) 4.76 (0.94–28.77), P-trend = 0.06, and 1.79 (0.61–5.29), P-trend = 0.37, for Pre-menopausal and Post-menopausal breast cancer, respectively).

**Table 3:** LIPG and breast cancer risk by menopausal status.

	Number cases/controls	OR^a^	95% CI	OR^b^	95% CI
**Pre-Menopausal**					
LIPG < 1.18 ng/ml	8/12	1.00		1.00	
LIPG 1.18–2.78 ng/ml	9/6	1.82	0.34–10.49	2.13	0.34–14.61
LIPG > 2.78 ng/ml	28/9	4.20	0.99–19.86	4.76	0.94–28.77
P-trend			0.057		0.060
**Post-Menopausal**					
LIPG < 1.18 ng/ml	15/11	1.00		1.00	
LIPG 1.18–2.78 ng/ml	12/17	0.52	0.17–1.51	0.45	0.13–1.50
LIPG > 2.78 ng/ml	35/14	1.83	0.67–5.00	1.79	0.61–5.29
P-trend			0.183		0.372

^a^Adjusted for age at diagnosis (cases) and age at interview (controls).

^b^Further adjusted for age at menarche, parity, BMI, and family history of breast/ovarian cancer.

Table 4 shows the LIPG-breast cancer association according to the main breast cancer subtypes. The association was restricted to Luminal A breast cancers (Multivariable OR for the highest category (95% CI) 3.70 (1.42–10.16), P-trend = 0.015, and 2.05 (0.63–7.22), P-trend = 0.311, for Luminal A and non-Luminal A breast cancers, respectively), but we did not have sufficient precision for other subtypes of breast cancer. Similarly, subset analysis only based on HER2 receptor status indicated that the LIPG-breast cancer relationship was restricted to HER2-negative breast cancers (Multivariable OR for the highest category (95% CI) 4.39 (1.70–12.03), P-trend = 0.012, and 1.10 (0.28–4.32), P-trend = 0.745, for HER2-negative and HER2-positive tumors, respectively).

**Table 4:** LIPG and breast cancer risk by subtypes.

	Number cases/controls	OR^a^	95% CI	OR^b^	95% CI
**Luminal A**					
LIPG < 1.18 ng/ml	12/23	1.0		1.0	
LIPG 1.18–2.78 ng/ml	16/23	1.29	0.50–3.40	1.38	0.47–4.17
LIPG > 2.78 ng/ml	40/23	3.25	1.38–7.98	3.70	1.42–10.16
P-trend			0.005		0.015
**Non luminal A**					
LIPG < 1.18 ng/ml	7/23	1.0		1.0	
LIPG 1.18–2.78 ng/ml	2/23	0.27	0.04–1.28	0.38	0.05–2.00
LIPG > 2.78 ng/ml	14/23	1.92	0.66–5.96	2.05	0.63–7.22
P-trend			0.229		0.311
**HER2 Positive**					
LIPG < 1.18 ng/ml	7/23	1.0		1.0	
LIPG 1.18–2.78 ng/ml	1/23	0.13	0.01–0.84	0.21	0.01–1.51
LIPG > 2.78 ng/ml	7/23	0.97	0.28–3.31	1.10	0.28–4.32
P-trend			0.881		0.745
**HER2 Negative**					
LIPG < 1.18 ng/ml	12/23	1.0		1.0	
LIPG 1.18–2.78 ng/ml	17/23	1.37	0.53–3.59	1.49	0.51–4.49
LIPG > 2.78 ng/ml	47/23	3.81	1.63–9.28	4.39	1.70–12.03
P-trend			0.004		0.012

^a^Adjusted for age at diagnosis (cases) and age at interview (controls).

^b^Further adjusted for age at menarche, parity, menopausal status, BMI, and family history of breast/ovarian cancer.

Table 5 shows the LIPG-breast cancer association stratified by total cholesterol levels. The LIPG-breast cancer association was restricted to women with high total cholesterol levels (Multivariable OR for the highest LIPG category (95% CI) 6.30 (2.13–20.05), P-trend = 0.018, and 0.65 (0.11–3.28), P-trend = 0.786, among women with high and low cholesterol levels, respectively).

**Table 5:** LIPG and risk of breast cancer by total cholesterol.

	Number cases/controls	OR^a^	95% CI	OR^b^	95% CI
**Cholesterol ≤ 188 mg/dl**					
LIPG < 1.18 ng/ml	11/7	1.00		1.00	
LIPG 1.18–2.78 ng/ml	5/5	0.61	0.11–3.31	0.51	0.05–4.78
LIPG > 2.78 ng/ml	17/11	0.74	0.18–2.81	0.65	0.11–3.28
P-trend			0.992		0.786
**Cholesterol > 188 mg/dl**					
LIPG < 1.18 ng/ml	12/16	1.00		1.00	
LIPG 1.18–2.78 ng/ml	16/18	1.20	0.44–3.35	1.30	0.41–4.18
LIPG > 2.78 ng/ml	46/9	6.91	2.51–20.41	6.30	2.13–20.05
P-trend			0.004		0.018

^a^Adjusted for age at diagnosis (cases) and age at interview (controls).

^b^Further adjusted for age at menarche, parity, menopausal status, BMI, and family history of breast/ovarian cancer.

Table 6 shows the effect of LIPG in non-postpartum and postpartum breast cancer (defined as a breast cancer diagnosis within 10 years of last childbirth) among parous women. The LIPG-breast cancer association was restricted to non-postpartum breast cancer (Multivariable OR for the highest category (95% CI) 3.83 (1.37–11.39), P-trend = 0.003, and 2.35 (0.16–63.65), P-trend = 0.396, for non-postpartum and postpartum breast cancer, respectively), although we lacked precision among postpartum breast cancer (there were only nine cases). We also examined the LIPG-breast cancer association by tumor grade and histology (Table 6). The LIPG-breast cancer association was found to be associated with grades II and III breast cancers (Multivariable ORs for the highest category of LIPG (95% CI) 2.73 (1.02–7.69), P-trend = 0.057, and 1.90 (0.61–6.21), P-trend = 0.170, for grades II and III, and grade I breast cancers, respectively). No differences were detected by histology (data not shown).

**Table 6:** LIPG and risk of breast cancer by time of diagnosis and grade.

	Number cases/controls	OR^a^	95% CI	OR^b^	95% CI
**Non-postpartum breast cancer**^**c**^					
LIPG < 1.18 ng/ml	12/15	1.00		1.00	
LIPG 1.18–2.78 ng/ml	8/19	0.53	0.16–1.67	0.40	0.10–1.45
LIPG > 2.78 ng/ml	41/16	3.60	1.35–10.04	3.83	1.37–11.39
P-trend			0.003		0.003
**Postpartum breast cancer**^**c**^					
LIPG < 1.18 ng/ml	1/15	1.00		1.00	
LIPG 1.18–2.78 ng/ml	3/19	3.64	0.31–94.49	3.34	0.25–96.25
LIPG > 2.78 ng/ml	5/16	4.37	0.45–102.46	2.35	0.16–63.65
P-trend			0.105		0.396
**Grade I**					
LIPG < 1.18 ng/ml	8/23	1.00		1.00	
LIPG 1.18–2.78 ng/ml	7/23	0.81	0.24–2.67	0.67	0.17–2.48
LIPG > 2.78 ng/ml	16/23	1.90	0.69–5.55	1.90	0.61–6.21
P-trend			0.166		0.170
**Grade II and III**					
LIPG < 1.18 ng/ml	12/23	1.00		1.00	
LIPG 1.18–2.78 ng/ml	11/23	0.91	0.33–2.49	1.17	0.38–3.68
LIPG > 2.78 ng/ml	30/23	2.43	1.01–6.06	2.73	1.02–7.69
P-trend			0.030		0.057

^a^Adjusted for age at diagnosis (cases) and age at interview (controls).

^b^Further adjusted for age at menarche, parity, menopausal status, BMI, and family history of breast/ovarian cancer.

^c^Among parous women only.

## Discussion

LIPG is critical for the acquisition of indispensable extracellular lipids that breast cancer cells need to be able to grow and proliferate^15^. LIPG increased risk of gastric and testis cancers in previous studies^13,14^. However, very limited data exists on the role of LIPG on the risk of breast cancer.

In the present study, we found increased levels of LIPG to be associated with risk of breast cancer, especially breast cancer subtypes Luminal A and HER2-negative. To our information, this is the first examination of plasma LIPG and breast cancer risk, overall and by major breast cancer subtypes. The association between LIPG and risk of breast cancer was more pronounced among women with total cholesterol levels higher than 188 mg/dl, and among grades II and III breast cancer.

One previous study examined LIPG expression levels in urine samples of stomach cancer patients and healthy volunteers^14^. There was approximately a tenfold average decrease in the LIPG expression levels in urine samples of stomach cancer compared to healthy individuals, producing an AUC of 0.967^14^. Plasma levels of LIPG may show, based on the results of this and previous studies, opposing effects on cancer, decreasing or increasing the risks, similar to what is seen with NLR and gastric and breast cancer in previous studies^21^.

Breast cancer cells need lipids to grow and LIPG is crucial for acquiring the indispensable extracellular lipids needed for breast cancer cells to grow and proliferate^15^. LIPG activity is essential for extracellular lipid uptake which is needed for subsequent proliferation of breast cancer cells. FoxA1 and the transcription factors family regulate the expression of LIPG^15^.

It has been shown that a decrease in LIPG inhibits breast cancer growth, implying that the incorporation of extracellular lipids, a function of LIPG, is crucial for the growth of cancer cells^15^. This is an important finding since it was amply thought that de novo fatty acid formation was the principal driver of tumor growth^22^. Laboratory data with breast cancer cells lacking LIPG showed a remarkable reduction of the majority of intracellular glycerol-lipid intermediates in the formation of triglycerides and their derivatives^15^. Several lipids and/or derivatives in the media were not reduced in LIPG-depleted cells as much as in untreated cells, therefore implying that extracellular lipids are the substrates for intracellular lipid formation^15^. Specifically, it was demonstrated the crucial role of extracellular lipid species for breast cancer cell growth in a lipoprotein-depleted medium, a process building upon LIPG^15^.

In concert with this finding, it has been shown that a high-fat diet was able to rescue the absence of monoacylglycerol lipase, an important intracellular lipase, for cancer pathogenesis, since cancer cells were able to uptake lipids from the extracellular compartment^15,23^. It has been shown that this rescue mechanism is not functional in breast cancer cells in the absence of LIPG or FoxA2^15^.

It has also been shown that LIPG activity releases fatty acids from HDL phospholipids and these fatty acids are further employed for intracellular lipid production in the human hepatic cell line HepG2^24,25^.

Breast cancer cells are dependent on a mechanism that allows them to extract lipid precursors from extracellular sources for intracellular lipid production, a process that is needed for cancer cells to be able to proliferate, and LIPG realizes this function^15^. Therefore, LIPG is a major player for lipid metabolic adaptations that breast cancer cells must undergo to continue proliferating^15^. De novo lipid synthesis is necessary but not sufficient to support lipid production needed for breast cancer tumor growth^15^. Consistent with this notion, previous studies have reported an association between circulating lipids and risk of breast cancer in women with extensive mammographic density^26^. This finding may suggest that interventions aimed to reduce circulating lipids may have an effect on breast cancer risk^26^. We have previously analyzed the effect of circulating lipids (total cholesterol, LDL, HDL), and breast cancer stratified by LIPG levels and found opposing effects by LIPG levels^27,28^. We found total cholesterol to increase risk of breast cancer at high levels of LIPG, but to decrease risk at low levels (OR (95% CI) for high levels of total cholesterol among women with high LIPG levels was 8.61 (95% CI 2.38–36.08) versus 0.15, (0.02–0.74) among women with low levels of LIPG (unpublished data)^28^. We previously found circulating triglyceride and total cholesterol levels to increase the risk of breast cancer^27,28^. Altogether, these observations make LIPG activity a crucial player for breast carcinogenesis, specially for lipids-induced breast carcinogenesis.

Cadenas and collaborators also contributed substantially to this topic. They reported that LIPG is increased during oxidative stress and increases survival of cells that are no longer able to generate a sufficient supply of fatty acids by de novo synthesis ^29^. Similar to Slebe et al.^15^, Riederer et al. ^30^ has also shown that LIPG releases free fatty acids which can be taken up by cells. It has also been shown that overexpression of an oncogenic form of HER2 leads to strong expression of LIPG^31^ and LIPG has been reported to be associated with tumor growth ^15^ and with metastasis in triple-negative breast cancer ^32^.

Cadenas and colleagues also showed that LIPG enables breast cancer cell lines to utilize circulating lipoproteins to synthetize and store triglycerides in lipid droplets ^29^. Furthermore, they showed that oxidative stress under conditions that block endogenous fatty acid synthesis induces LIPG expression and activity. A critical finding of the Cadenas et al.^29^ study is that LIPG upregulation protects the cancer cells from mitochondrial dysfunction and cell death. Cadenas et al., also showed that a small fraction of tumors overexpresses LIPG which was associated with shorter metastasis-free survival. To summarize, LIPG upregulation seems to be one of the mechanisms how cancer cells can guarantee fatty acid supply from extracellular sources under conditions where oxidative stress blocks endogenous synthesis.

We believe that LIPG is an active player in breast carcinogenesis for the following reasons. (1) LIPG upregulation protects cancer cells from mitochondrial dysfunction and cell death, and (2) LIPG increases survival of cancer cells that are no longer able to generate a sufficient supply of fatty acids by de novo synthesis, in a crucial survival mechanism for cancer cells. In summary, breast cancer cells need lipids to grow and proliferate and LIPG is instrumental in proportioning them to the cancer cells.

LIPG is also the major lipase of the TLG family present in the human placenta^33^, and is dysregulated in conditions such as intrauterine growth restriction^33^. In placentas from other pregnancy-related conditions, such as obese GDM pregnancies, LIPG expression was also upregulated by 1.9-fold compared with lean pregnancies^33^. We examined the effect of LIPG in postpartum and non-postpartum breast cancer. The LIPG-breast cancer association was restricted to non-postpartum breast cancer (Multivariable OR for the highest LIPG category (95% CI) 3.83 (1.37–11.39), P-trend = 0.003, and 2.35 (0.16–63.65), P-trend = 0.396, for non-postpartum and postpartum breast cancer, respectively), although we lacked precision for postpartum breast cancer.

We also examined plasma levels of OxLDL and risk of breast cancer. In previous studies, serum levels of OxLDL are increased in pregnant compared to non-pregnant women^34^. OxLDL is also higher in preeclampsia compared to normal controls^35^. In experimental studies, OxLDL activate both apoptosis and autophagy in cancer cells^36^. However, in the present study, we found no increased breast cancer risk from OxLDL, although we lacked precision.

Because of the LIPG-HDL interrelationship, we also examined the HDL-breast cancer association. Experimental studies have shown that HDL-C can enhance cellular proliferation of breast cancer cell^37,38^ supporting the positive association between HDL-C and breast cancer risk observed in previous studies^39^. However, we did not find increased or decreased breast cancer risk associated for HDL levels in the present study (data not shown).

### Study limitations

The present study has several limitations. The first limitation is the small sample size which reduces the power to conduct meaningful stratified analyses. Our study also lacked pre-diagnostic samples from the patients to be able to conclude that LIPG increases the risk of breast cancer. We also lacked sufficient follow-up data on survival or recurrence of breast cancer, precluding the examination of the LIPG-breast cancer association on survival or recurrence. On the other hand, a strength of our study is the available information on HER2 receptor status, in conjunction with ER and PR receptor status.

The LIPG levels reported by different studies to date are quite discordant. The LIPG (Endothelial lipase, EL) ELISA Kit was purchased from CUSABIO as stated in the Material and Methods section. This Kit specifically detects plasma levels of LIPG with a detection range of 0.625–40 ng/ml. There are several kits for LIPG detection with different ranges of sensitivity, i.e., LSBIO (0.156–10 ng/ml); Antibodies (0.078–5 ng/ml); MyBioSource (0.1–2.5 ng/ml), and TaKaRa/Immuno-Biological-Laboratories (0.031–2 ng/ml). There are few studies on plasma levels of LIPG in cancer and other pathologies, and they are quite discordant, with mean values ranging from 0.05 to 420 ng/ml ^40–45^. More studies are needed to establish the “normal” range of LIPG levels. All LIPG values in our study were under the maximum detection level specified by the provider, and both intra-assays and inter-assays precision was with a CV < 10%.

We found a more pronounced association among Luminal A breast cancers. As Luminal A and other breast cancer subtypes are treated with different chemotherapy treatments, it is tempting to think that the different effect of LIPG in Luminal A cancers, may be the result of the treatment used to treat that specific subtype of breast cancer. However, all our breast cancer patients were studied, and their sample taken, before any cancer diagnosis, therefore before any cancer treatment, chemotherapy or any other type. Thus, although Luminal A and other breast cancer subtypes are treated with different treatments, it is not possible that any more pronounced effect of LIPG on the risk of Luminal A tumors was due to treatment differences.

Because of the same reason, i.e., that all our samples were collected before cancer diagnosis, it is not likely that tumor-secreted LIPG is responsible for high plasma LIPG levels, or that increased LIPG reflects tumor-induced increased inflammatory state in our patients. However, there is still a possibility that early stages of a latent tumor may have caused the increased LIPG levels, although it is very small. In addition, since we lacked information on CRP or IL-6, further studies with these markers may shed light in the LIPG-breast cancer association.

## Conclusions

To our knowledge, this is the first study examining the association between circulating LIPG and breast cancer overall and by subtypes. Our findings indicate that elevated LIPG levels are associated with increased risk of breast cancer, especially Luminal A and HER2-negative breast cancers. The LIPG-breast cancer association appeared to be restricted or more pronounced among women with non-postpartum breast cancer, those with high levels of total cholesterol, and those with grades II and III breast cancers.

Experimental studies have shown that, to be able to grow, breast cancer cells need to get lipids from extracellular sources and LIPG is in charge of this^15^. This means that LIPG activity is essential for lipid uptake which is needed for subsequent proliferation of breast cancer cells^15^. LIPG could be a new target for chemoprevention and treatment of breast cancer.

## Material and methods

### Study population

The Breast Oncology Galician Network (BREOGAN) study is a population-based case–control study, including 1766 cases and 1205 controls, conducted in the cities of Santiago de Compostela and Vigo, Spain, within a geographically defined health region that covers approximately one million inhabitants. Data collection methods have been previously described^17–21,46,47^. BREOGAN counts with 1766 women with invasive breast cancer diagnosed and treated between 1997 and 2014 at the Clinical University Hospitals of Santiago (CHUS) and Vigo (CHUVI). Controls are 1205 women living in the same population health area as cases free of cancer, except non-melanoma skin cancer. 114 breast cancer cases and 82 controls had data on LIPG and/or OxLDL and were included in the present study. Response rates were 98% and 99% for cases and controls, respectively. Ethics approval for this study was obtained from the Galician Ethics and Research Committee (CEIC, Comité Ético de Investigación Clínica de Galicia), responsible for the oversight of both university hospitals, CHUS and CHUVI, and family clinics from where all participants were recruited. All participants provided written informed consent. The study was conducted in accordance to the Helsinki Principles of 1975, as revised in 1983.

### Data collection

#### Risk factor data

Similar to previous studies^18,21^ risk factor information was collected through a risk factor questionnaire adapted from the *Ella* Binational Breast Cancer Study^17,48,49^ to meet the needs of the population in Spain. Clinical and histopathological information was abstracted from computerized medical records by trained physicians. The following variables were recorded: level of education [uneducated (less than primary education), primary education, secondary education, vocational training, 3-years degree (certificate, middle engineering), 5-years degree (graduate school, bachelor´s degree, superior engineering), and PhD (doctorate)], lifetime breastfeeding [categorized as no breastfeeding, < lifetime breastfeeding duration (7 months), ≥ lifetime breastfeeding duration (7 months)], age at menarche (categorized as ≤12, >12), age at first full-term pregnancy, parity (categorized as never vs. ever pregnant), age at diagnosis, age at menopause (categorized as ≤50, >50), menopausal status at diagnosis (categorized as pre and post-menopausal), number of pregnancies (categorized as none, 1–2, ≥ 3), oral contraceptive use (never, ever), hormone replacement therapy (HRT) (never, ever), body mass index (BMI) (<25, 25-29, ≥30), smoking status (never smoker, ex-smoker, current smoker), family history (categorized as none vs. one or more first and/or second-degree relatives with breast and/or ovarian cancer).

#### Clinic-pathological data

Similar to previous studies^17–21^ histopathological information was abstracted from computerized medical records by trained physicians. Immunohistochemistry (IHC) analyses on paraffin-embedded material have been previously performed following standard procedures in Galician hospitals to determine the status of ER and PR. In every tumor, 4-μm histological sections were cut and stained with hematoxylin and eosin for histopathological examination according to the criteria of the World Health Organization^50^. Histological grading was evaluated using the Nottingham modification of the Bloom–Richardson system^51^.

IHC analysis on paraffin-embedded material, as described in our previous study^18^, was performed using a universal second antibody kit that used a peroxidase-conjugated labeleddextran polymer (EnVision, Peroxidase/DAB; Dako, Glostrup, Denmark), with antibodies for ER (clone 6F11, dilution 1:50, water bath; Novocastra, Newcastle-upon- Tyne, UK), PR (clone PgR 636, dilution 1:50, water bath; Dako, Glostrup, Denmark)^18^. Negative and positive controls were concurrently run for all antibodies with satisfactory results. Cells were considered immunopositive when diffuse or dot-like nuclear staining was observed regardless of the intensity of the staining; only nuclear immunoreactivity was considered specific. The number of positive cells was counted by two different observers independently. Whenever necessary, a consensus was reached using a double-headed microscope. ER and PR were considered positive when the percent of immunostained nuclei was ≥ 10%.

Similar to previous studies^17–21^, imunohistochemistry (IHC) analyses were performed to determine HER2 status (Dako). No immunostaining (0) or weak membrane immunostaining (1+) was considered low HER2 expression (HER2). Strong membrane immunostaining (3+) was considered HER2 overexpression (HER2+). Moderate membrane staining (2+) samples were further analyzed using fluorescence in situ hybridization techniques; they were considered to be HER2+ if the ratio of cerb-B2/centromere 17 copy number was > 2.0.

Similar to previous studies^17–21^, ER, PR and HER2 status (categorized as positive and negative), grade (categorized as I—well differentiated, II—moderately differentiated and III—poorly differentiated or undifferentiated), histology type (categorized as invasive ductal carcinoma, invasive lobular carcinoma and other), and tumor size (mm). As previously described in our studies^18,21^, of the 1766 women who participated in the study, 100 had unknown ER status, 114 had unknown PR status, and 340 had unknown HER2 status. One hundred and eighty-four women had unknown grade, 14 had unknown histological type and 144 had unknown tumor size^18,21^. Sixty-two women had unknown age at menarche, and 48, out of 1443 parous women, had unknown lifetime breastfeeding^18,21^.

Plasma endothelial lipase (LIPG) was measured by ELISA test using the Human Endothelial Lipase, EL ELISA kit from Cusabio (CSB-E08217h), and Oxidized LDL (OxLDL) was measured by ELISA test using the Human Oxidized LDL kit from Mercodia (10-132-01). The ELISA tests are solid phase two-side enzyme immunoassays based on the direct sandwich technique.

### Statistical analyses

The association of breast cancer with LIPG and OxLDL was measured by odds ratios (ORs) and corresponding 95% confidence intervals (CIs) using polytomous logistic regression. Similar to previous studies^21^, analyses were initially adjusted for the following established risk or protective factors for breast cancer: reference age (age at diagnosis for cases and age at interview for controls), age at menarche, parity, breastfeeding, menopausal status, weight, height, and family history of first and/or second-degree relatives with breast and/or ovarian cancer. Results were virtually unchanged after adjustment for all these variables or only age, age at menarche, parity, menopausal status, BMI, and family history of first and/or second-degree relatives with breast and/or ovarian cancer, therefore we present results adjusted for the latter. Outcome (dependent) variables were breast cancer subtypes defined by ER, PR, and HER2 status (we defined four tumor subtypes (ER+/HER2− or PR+/HER2− [Luminal A], ER+/HER2+ or PR+/HER2+ [Luminal B], ER−/PR−/HER2+ [HER2 overexpressing or HER2+], and ER−/PR−/HER2−[TNBC])), compared to controls (comparison group), and explanatory variables were LIPG and OxLDL. Cutoff points for subgroup analysis, i.e., LIPG, OxLDL and total cholesterol, were calculated based on distribution among controls. Cut points for serum total cholesterol levels were based on tertile distribution among the control group (≤ 188, > 188), which are equivalent to standard levels of normal and borderline/high total cholesterol levels (≤  200 mg/dl, > 200 mg/dl). All statistical analyses were performed using the R statistical software version 3.3.3. All reported test significance levels (P values < 0.05) were two-sided.
